# Understanding CT imaging findings based on the underlying pathophysiology in patients with small bowel ischemia

**DOI:** 10.1007/s11604-022-01367-x

**Published:** 2022-12-06

**Authors:** Yuko Nakamura, Shota Kondo, Keigo Narita, Shogo Maeda, Dara Fonseca, Yukiko Honda, Chihiro Tani, Wataru Fukumoto, Hidenori Mitani, Mana Ishibashi, Keigo Chosa, Fuminari Tatsugami, Kazuo Awai

**Affiliations:** Diagnostic Radiology, Hiroshima University, 1-2-3 Kasumi, Minami-Ku, Hiroshima, 734-8551 Japan

**Keywords:** Small bowel ischemia, Computed tomography, X-ray, Small bowel obstruction, Mesenteric ischemia

## Abstract

Because acute small bowel ischemia has a high mortality rate, it requires rapid intervention to avoid unfavorable outcomes. Computed tomography (CT) examination is important for the diagnosis of bowel ischemia. Acute small bowel ischemia can be the result of small bowel obstruction or mesenteric ischemia, including mesenteric arterial occlusion, mesenteric venous thrombosis, and non-occlusive mesenteric ischemia. The clinical significance of each CT finding is unique and depends on the underlying pathophysiology. This review describes the definition and mechanism(s) of bowel ischemia, reviews CT findings suggesting bowel ischemia, details factors involved in the development of small bowel ischemia, and presents CT findings with respect to the different factors based on the underlying pathophysiology. Such knowledge is needed for accurate treatment decisions.

## Introduction

Acute small bowel ischemia is an abdominal emergency that accounts for about 2% of gastrointestinal illnesses. Due to the aging of the population and the consequently growing rate of degenerative vascular diseases, its incidence has increased [1]. Acute small bowel ischemia, a complicated disorder attributable to the interruption of the vascular flow to the small bowel, can be induced by mesenteric arterial occlusion, mesenteric venous thrombosis, non-occlusive mesenteric ischemia, and strangulated small bowel obstruction. As its mortality rate is high (50 to 80%) [2], acute small bowel ischemia requires rapid intervention to avoid unfavorable outcomes. However, clinical and laboratory signs of bowel strangulation lack the sensitivity for predicting bowel ischemia [3]. On the other hand, imaging studies provide valuable treatment guidance. Computed tomography (CT) is the most common imaging modality in the urgent setting because it is widely available, fast, and easy; the findings are highly reproducible and objective. As CT yields important information for making accurate treatment decisions, it is the preferred technique for the evaluation of small bowel ischemia [4].

CT findings suggestive of bowel ischemia can be specific or non-specific. As small bowel ischemia can be attributable to small bowel obstruction or mesenteric ischemia and CT findings depend on the underlying factors and pathophysiology, they must be known for the accurate interpretation of CT images.

This review describes the definition and mechanism(s) of bowel ischemia, reviews CT findings suggesting small bowel ischemia based on its mechanism(s), and provides insight into factors involved in the development of small bowel ischemia by referring to specific CT findings. Such knowledge is needed for accurate treatment decisions.

## Definition and mechanism(s) of bowel ischemia

### What is bowel ischemia?

Bowel ischemia includes potentially life-threatening conditions that reduce the blood flow to the bowel. Acute bowel ischemia is characterized by three stages that reflect the extent of bowel-wall involvement. At stage I, the disease is reversible and pathologically characterized by necrosis, erosion, ulceration, edema, and hemorrhage localized to the mucosa. In stage II, necrosis extends into the submucosal and muscularis propria layers. In stage III, transmural necrosis involves all three layers [5]. The mortality rate of patients with stage III bowel ischemia is high and an accurate diagnosis is imperative [6].

### Mechanisms of bowel ischemia (Fig. 1)

**Fig. 1:**
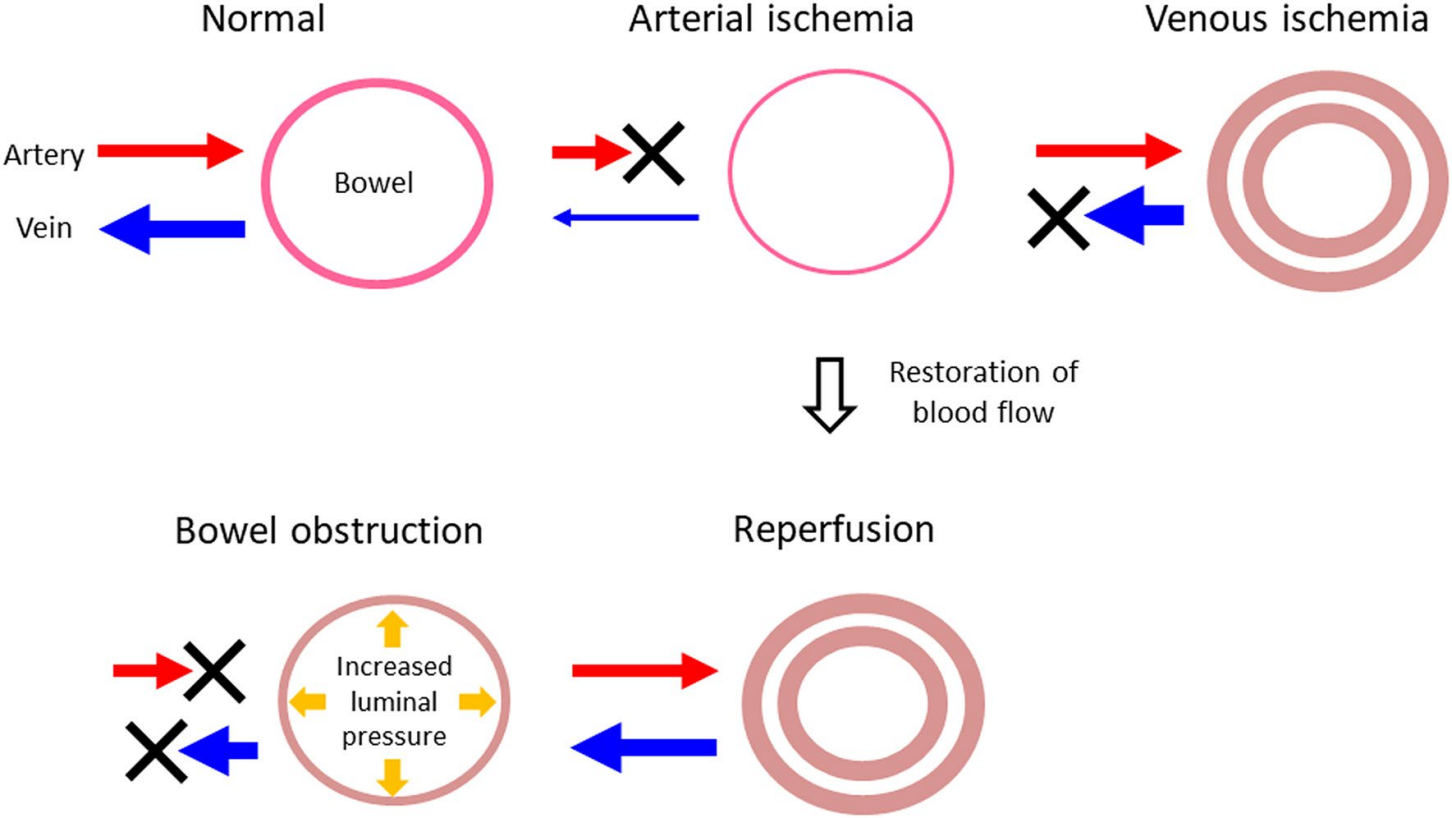
Schema of mechanisms implicated in bowel ischemia. Bowel-wall thinning is observed in patients with arterial ischemia; in the presence of venous ischemia, the bowel wall is thickened. After reperfusion, bowel-wall thickening is also observed in patients with arterial ischemia. The presence of venous obstruction can help differentiate between venous ischemia and reperfusion. In patients with bowel obstruction, the luminal pressure rises and initially exceeds the venous flow and subsequently the arterial flow, resulting in bowel ischemia

#### Inflow obstruction resulting in arterial ischemia

Arterial or venous obstruction can result in bowel ischemia. In the presence of arterial occlusion eliciting ischemia or transmural infarction, the small bowel is dilated and presents a classic “paper-thin wall” appearance attributable to a loss of bowel-wall tissue, the vasculature, and the muscle tone due to nerve and muscle damage [5].

#### Reperfusion

As an interruption in the blood supply results in ischemic injury that rapidly damages metabolically active tissues including bowel tissue, effective reperfusion by re-establishing the blood flow is critical to salvage ischemic tissues. Ineffective reperfusion, on the other hand, can lead to ischemia–reperfusion injury [7] and exacerbate the situation when the initial damage was due to advanced ischemia. Patients may experience shock, multiple organ failure, and they may die.

#### Outflow obstruction leading to venous ischemia

Venous obstruction increases the hydrostatic pressure on the bowel wall because high-pressure arterial inflow may persist despite venous occlusion. Vascular engorgement and edema of the bowel wall lead to extravascular fluid leakage into the bowel wall and mesentery. Venous drainage impairment ultimately results in the loss of the arterial supply and subsequent ischemia and infarction. Mesenteric edema and extravascular fluid tend to be seen more often in venous than in arterial ischemia [5].

#### Bowel ischemia due to bowel obstruction

Among patients with small bowel obstruction, 10% develop bowel ischemia [5] because the segment of the tract proximal to the obstruction site is dilated and filled with secretions and air. As the luminal content is trapped in the dilated intestine, its absorptive function is lost, leading to even more fluid sequestration. The luminal pressure increases to exceed first the venous flow and then the arterial flow, resulting in bowel ischemia [8].

## CT scanning and reconstruction protocol for bowel ischemia

Imaging studies are important for the evaluation of bowel ischemia. Multidetector CT (MDCT) is the most sensitive and specific diagnostic tool for bowel ischemia; it should be the first-line imaging modality. MDCT findings can help to exclude other factors producing acute abdominal pain. To include the entire course of the intestine, CT images should be acquired from the dome of the liver to the level of the perineum.

Both non-enhanced and biphasic contrast-enhanced CT images are needed [9]. Nonionic iodinated contrast material (600 mgI/kg) is delivered at a rate of 2.5–4 ml per second at a scanning delay of 30 s for the arterial phase and at 60–70 s for the venous phase. The CT slice thickness should not exceed 5 mm; for the precise evaluation, for multiplanar image reformation, and for CT angiography, the optimal thickness is 1–2 mm. Because the abdominal region is an inherently lower contrast area [10], an increase in the image noise adversely affects the image quality. Therefore, to reconstruct low-noise images, the standard radiation dose should be applied. If available, iterative- or deep-learning-based reconstruction is recommended for image-noise reduction [11–16]. For contrast-enhanced CT studies, sagittal images are useful for identifying the origin of the mesenteric arteries from the aorta and its variations. Coronal sections should be obtained routinely in addition to trans-axial sections. Arterial-phase maximum intensity projection (MIP) images should also be generated when mesenteric artery occlusion is suspected; venous-phase MIP images are needed when mesenteric vein occlusion is suspected [9, 17].

Abnormal bowel enhancement confirms the suspicion of bowel ischemia. While conventional single-energy CT relies on a single-energy spectrum to obtain attenuation-based images, dual-energy CT (DECT) facilitates material decomposition such as iodine quantification by acquiring two sets of images of the same body site by applying different photon spectra (high and low kV). Because DECT allows the accurate estimation of contrast enhancement based on iodine quantification on an iodine map, it is preferable to conventional single-energy CT for the evaluation of bowel enhancement in patients suspected of bowel ischemia [18–20].

## CT findings suggesting bowel ischemia (Table 1)

**Table 1:** CT imaging findings suggesting bowel ischemia

	Findings	Mechanisms in bowel ischemia
Bowel wall	Thinning (paper-thin wall)	Arterial ischemia
	Thickening	Reperfusion, venous ischemia
Bowel attenuation at unenhanced CT	High density (due to hemorrhage and hemorrhagic infarction)	All ischemia
Bowel enhancement	Absence	All ischemia
	Hyper-enhancement	Reperfusion, venous ischemia, early bowel obstruction
	Prolonged enhancement	Bowel obstruction (due to a reduction in the arterial perfusion and venous outflow)
Pneumatosis intestinalis	Air in the bowel wall	All ischemia
Mesenteric stranding	Increased attenuation of the mesenteric fat	Often seen in reperfusion and venous ischemia Transmural infarction in arterial ischemia and strangulated small bowel obstruction
Others	Ascites	Often seen in reperfusion and venous ischemia
Smaller SMV sign	Diameter of the SMV smaller than of the SMA	Arterial ischemia

*SMV* superior mesenteric vein, *SMA* superior mesenteric artery

### General findings

#### Bowel wall attenuation

**Fig. 2:**
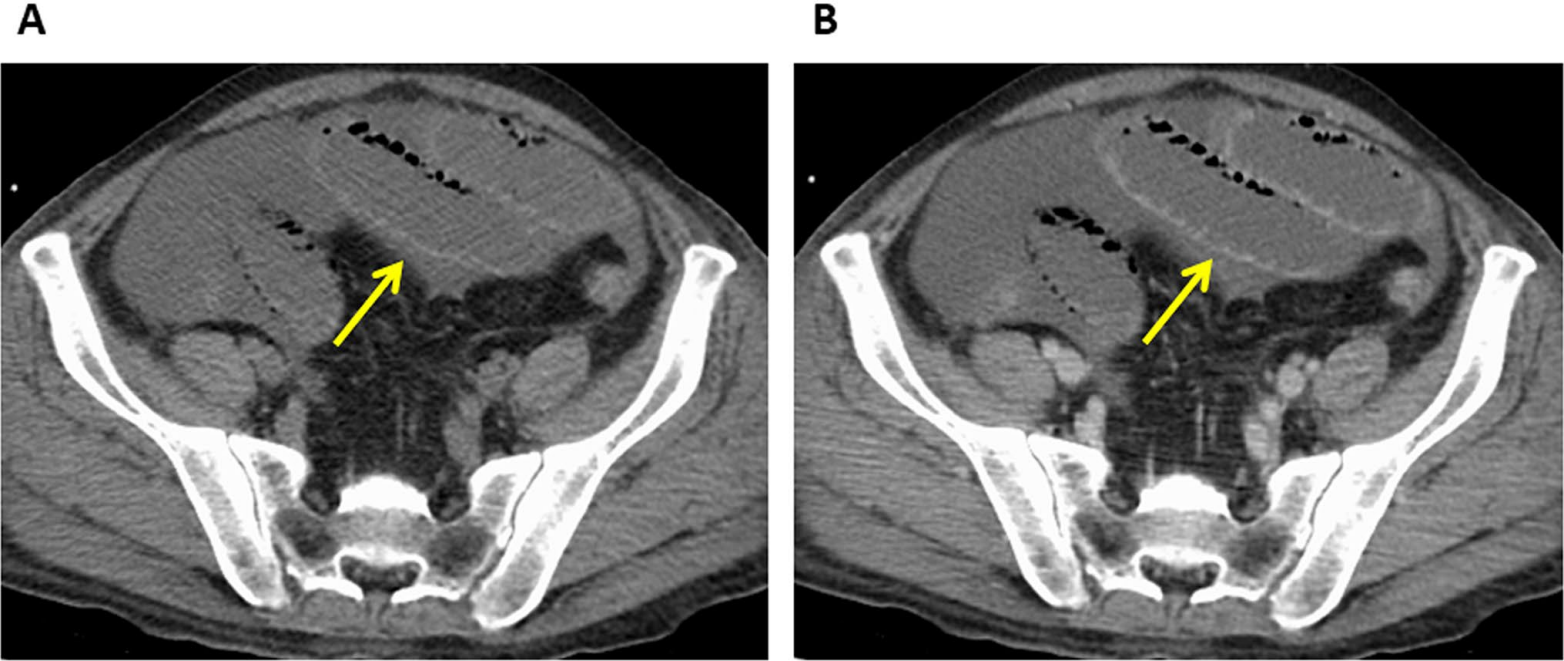
Non-occlusive mesenteric ischemia in a 50-year-old man with a history of alcoholic liver failure. **A.** Unenhanced CT image. The bowel wall is highly attenuated (arrow). **B.** Contrast-enhanced CT image. The wall seems to be well enhanced due to high attenuation (arrow). The unenhanced and enhanced images must be compared for an accurate assessment of bowel-wall contrast enhancement

**Fig. 3:**
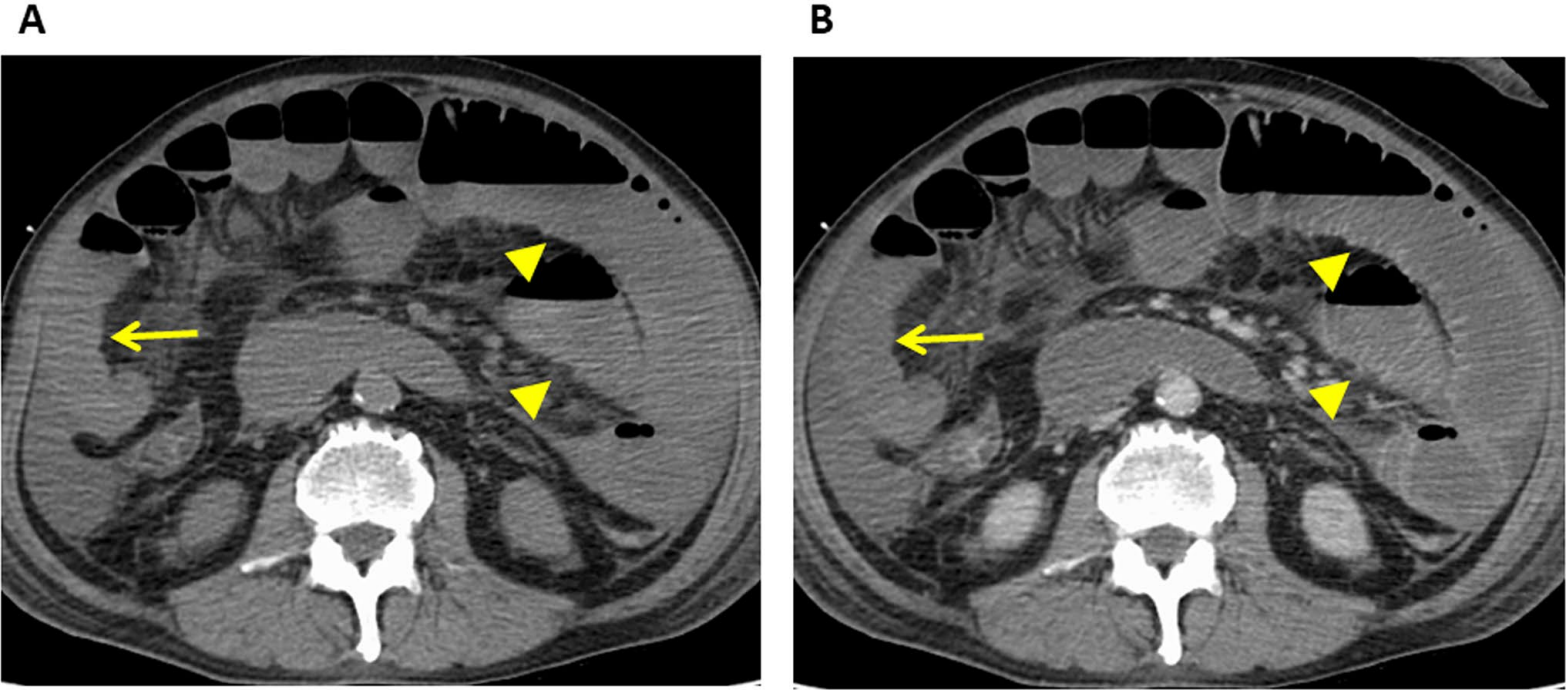
Non-occlusive mesenteric ischemia in a 50-year-old man (same patient as in Fig. 2). **A** Unenhanced CT image, **B** Contrast-enhanced CT image. The CT images show that the involved small bowel segments (arrows) are not contrast enhanced while the uninvolved segments are contrast enhanced (arrow heads)

**Fig. 4:**
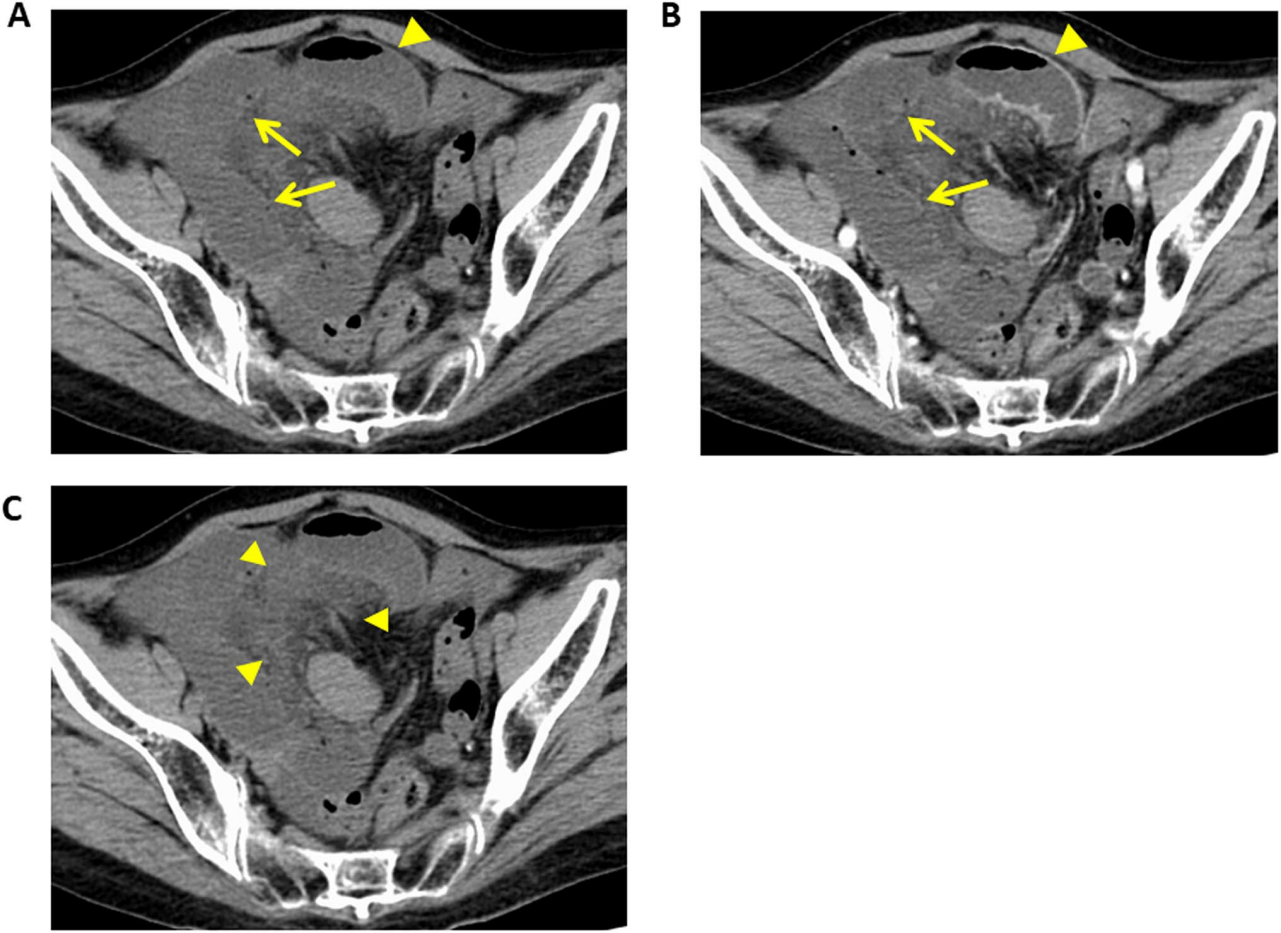
Strangulated small bowel obstruction due to an incarcerated hernia in a 69-year-old woman. **A** Unenhanced CT image, **B** Contrast-enhanced CT image, **C** Unenhanced CT image (same image as in 4A). **A**, **B** The CT images show that the involved (strangulated) small bowel segments (arrows) are not contrast enhanced while the uninvolved segments are contrast enhanced (arrow heads). **C** Mesenteric stranding (arrow heads) is seen

On unenhanced CT images, the affected bowel wall is highly attenuated due to intramural hemorrhage and hemorrhagic infarction [21] (Fig. 2). The absence of wall enhancement is a specific finding that indicates cessation of the arterial flow (Figs. 3 and 4). Unless the blood flow is restored, the bowel wall will become infarcted and perforated [22–24]. Therefore, the degree of bowel enhancement is important when there is a suspicion of bowel ischemia. Bowel-wall attenuation must be assessed on both unenhanced and contrast-enhanced CT images to avoid misinterpreting the high density of the bowel wall as normal positive enhancement on contrast-enhanced CT images of patients with intramural hemorrhage [9] (Fig. 2). When available, DECT scans should be acquired since their iodine maps may clearly demonstrate the ischemic area of the bowel [18–20].

#### Pneumatosis intestinalis

**Fig. 5:**
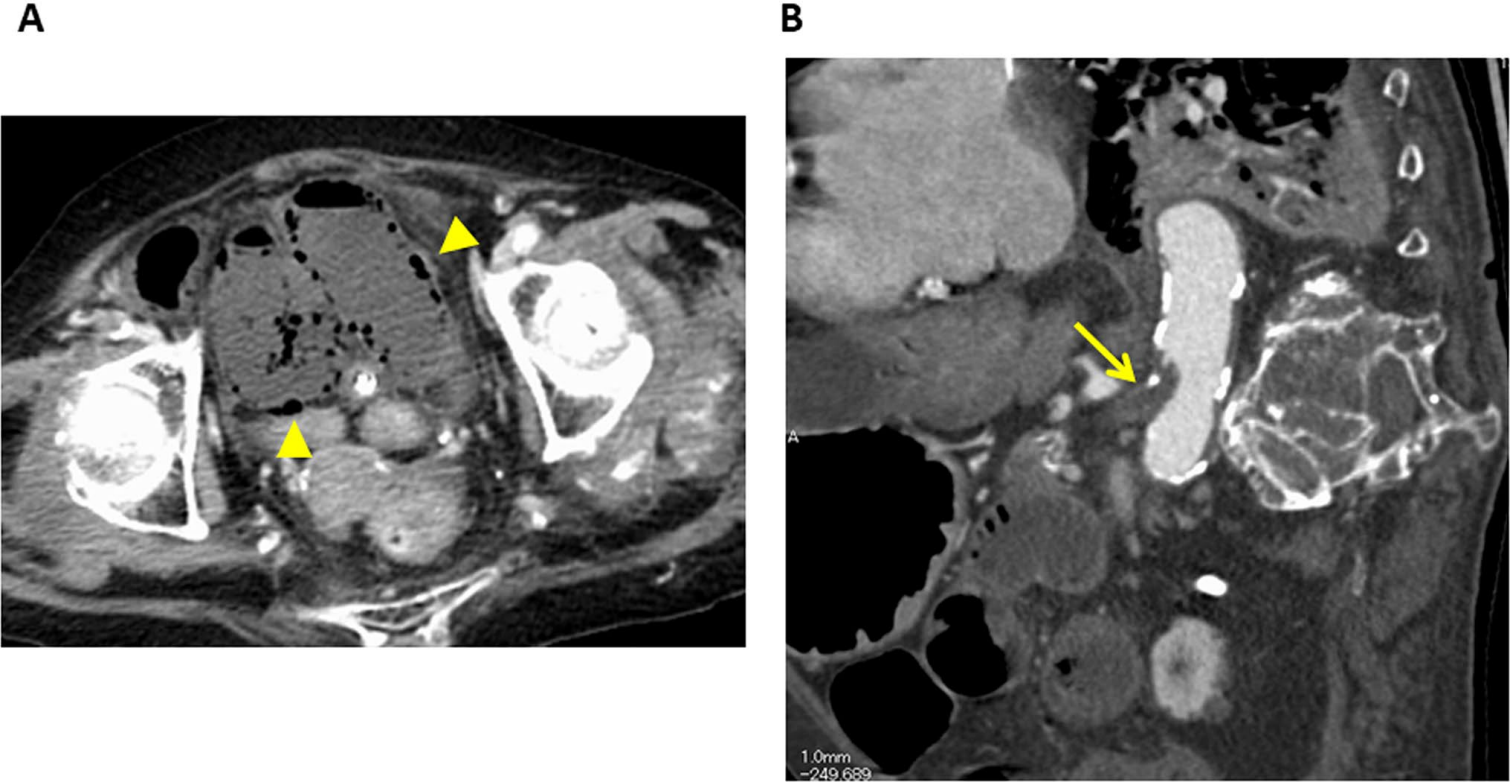
Superior mesenteric artery embolism in an 80-year-old woman with a history of a thrombus in the auricle of the left atrium. **A** Axial contrast-enhanced CT image (venous phase) revealing gas in the bowel wall (arrow heads) (pneumatosis intestinalis). **B** Oblique sagittal contrast-enhanced CT image (arterial phase) shows an occluding embolus (arrow) in the superior mesenteric artery

**Fig. 6:**
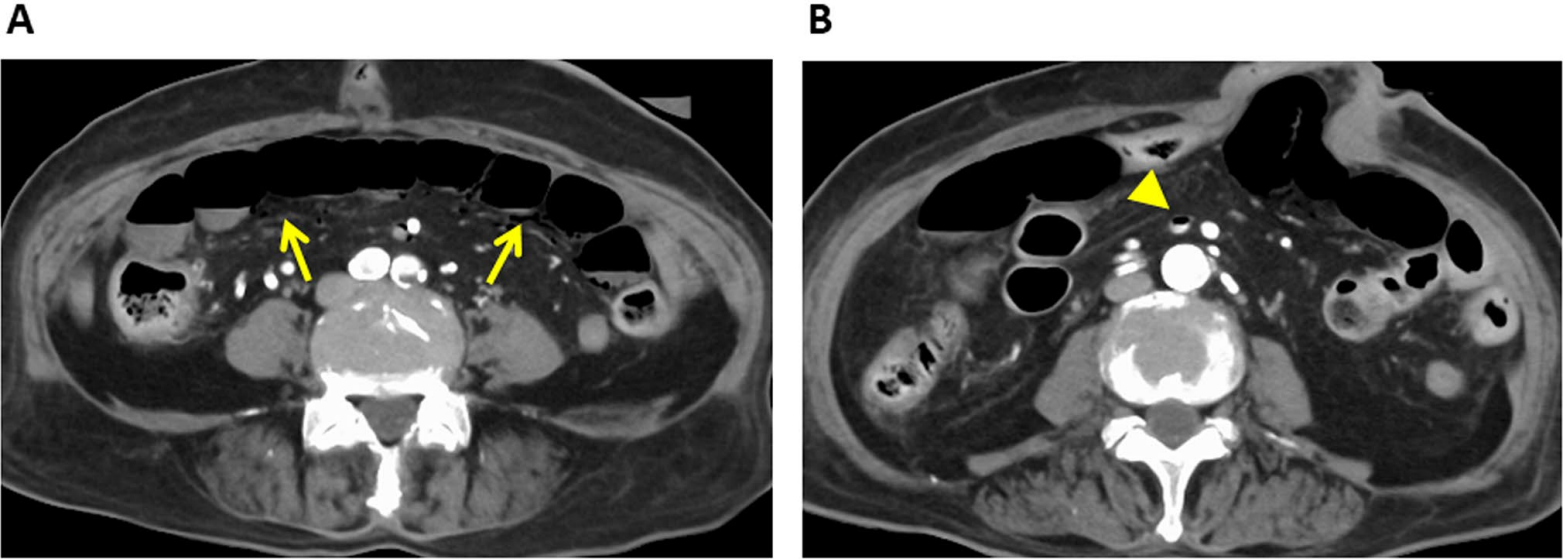
Non-occlusive mesenteric ischemia in an 87-year-old woman on dialysis for chronic renal failure. **A** Axial contrast-enhanced CT image shows thinning of the bowel wall (arrows) resulting in its paper-thin appearance. **B** Gas is seen in the superior mesenteric vein (arrow head)

**Fig. 7:**
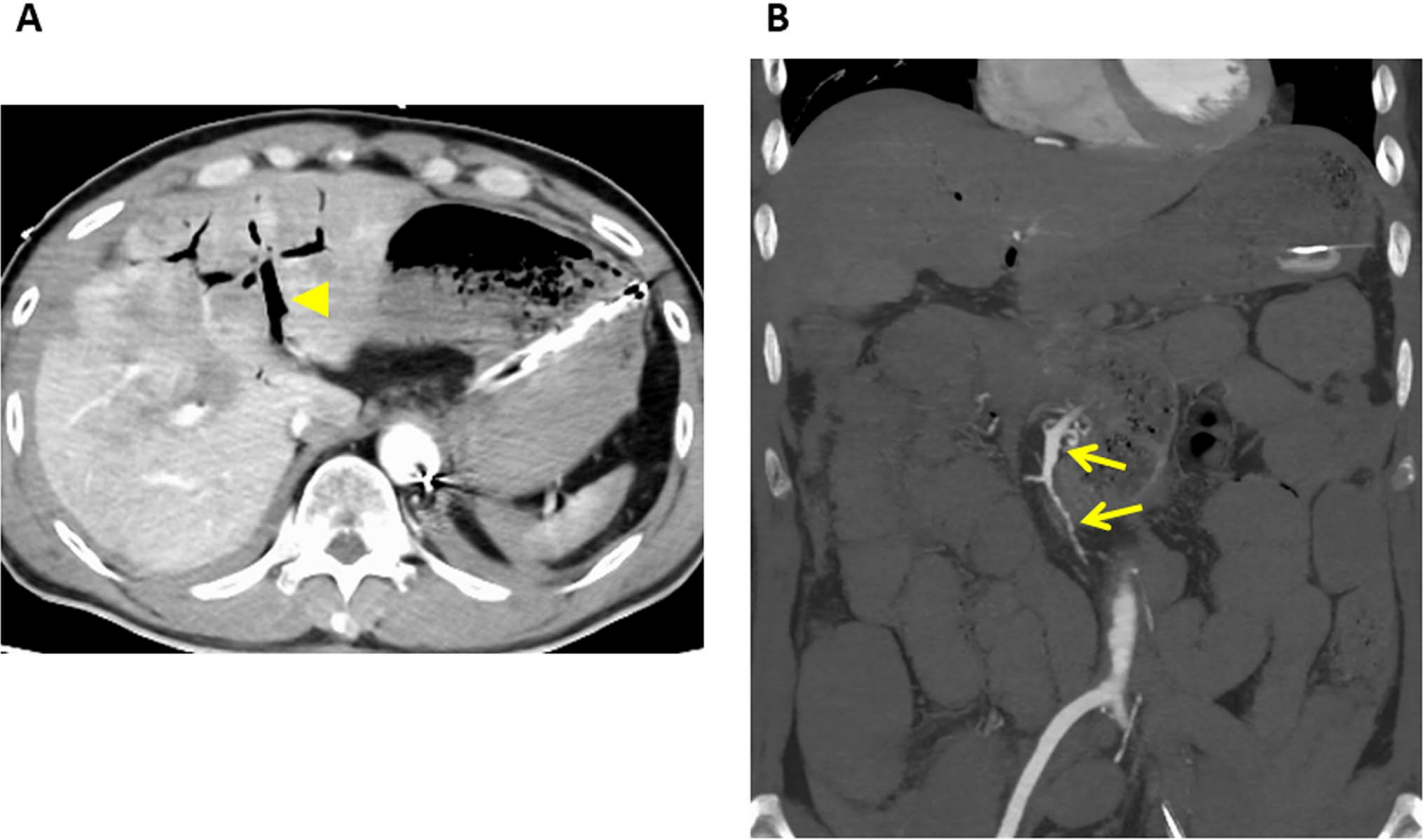
Non-occlusive mesenteric ischemia in a 73-year-old man with a history of polytrauma. **A** Axial contrast-enhanced CT image (venous phase) revealing gas in the intrahepatic portal vein (arrow head). **B** Coronal contrast-enhanced maximum intensity projection image (arterial phase). The superior mesenteric artery and its branches (arrows) show diffuse spasm

Air in the bowel wall on CT scans is indicative of pneumatosis intestinalis (Fig. 5); it often points to transmural infarction, especially when it is associated with portomesenteric venous gas (Figs. 6 and 7). However, pneumatosis intestinalis can also be encountered when the condition is benign and requires no intervention. Benign pneumatosis intestinalis is visualized as intramural cystic/bubble-like gas collections on CT images [25]. Worrisome features indicative of fulminant pneumatosis intestinalis include a linear or circumferential morphology of the intramural gas, bowel dilation, bowel-wall thickening, mesenteric stranding, hemorrhagic ascites, small bowel involvement, obstruction, ischemia, visceral infarction, portomesenteric venous gas, and perforation [26, 27]. Benign pneumatosis intestinalis is asymptomatic. Thus, not only imaging but also clinical findings should be considered to differentiate benign from worrisome pneumatosis intestinalis [28].

### Findings due to arterial ischemia

In patients with arterial bowel ischemia, the involved bowel wall may be thin. Its paper-thin appearance is attributable to a loss in the volume of tissues and vessels and to the loss of the intestinal muscle tone in patients with serious arterial occlusive ischemia or bowel infarction (inflow obstruction) (Fig. 6). Mesenteric stranding, increased attenuation of mesenteric fat, and ascites may be more frequent in venous than in arterial ischemia [5]. However, in patients with arterial occlusive mesenteric ischemia without reperfusion, this finding can help to estimate the severity of bowel ischemia because fat stranding on CT scans is almost exclusively seen in transmural infarction [9].

### Findings due to reperfusion

Bowel-wall thickening can be seen in the presence of arterial occlusion after reperfusion [9, 29]. On unenhanced CT images, attenuation is low in the presence of edema and high due to hemorrhage or associated infection. The thickened wall may have a target or a halo appearance on contrast-enhanced CT images; hyperenhancement can be seen due to hyperperfusion [9, 22]. Mesenteric stranding and ascites are non-specific findings attributable to reperfusion [29, 30].

### Findings due to venous ischemia

**Fig. 8:**
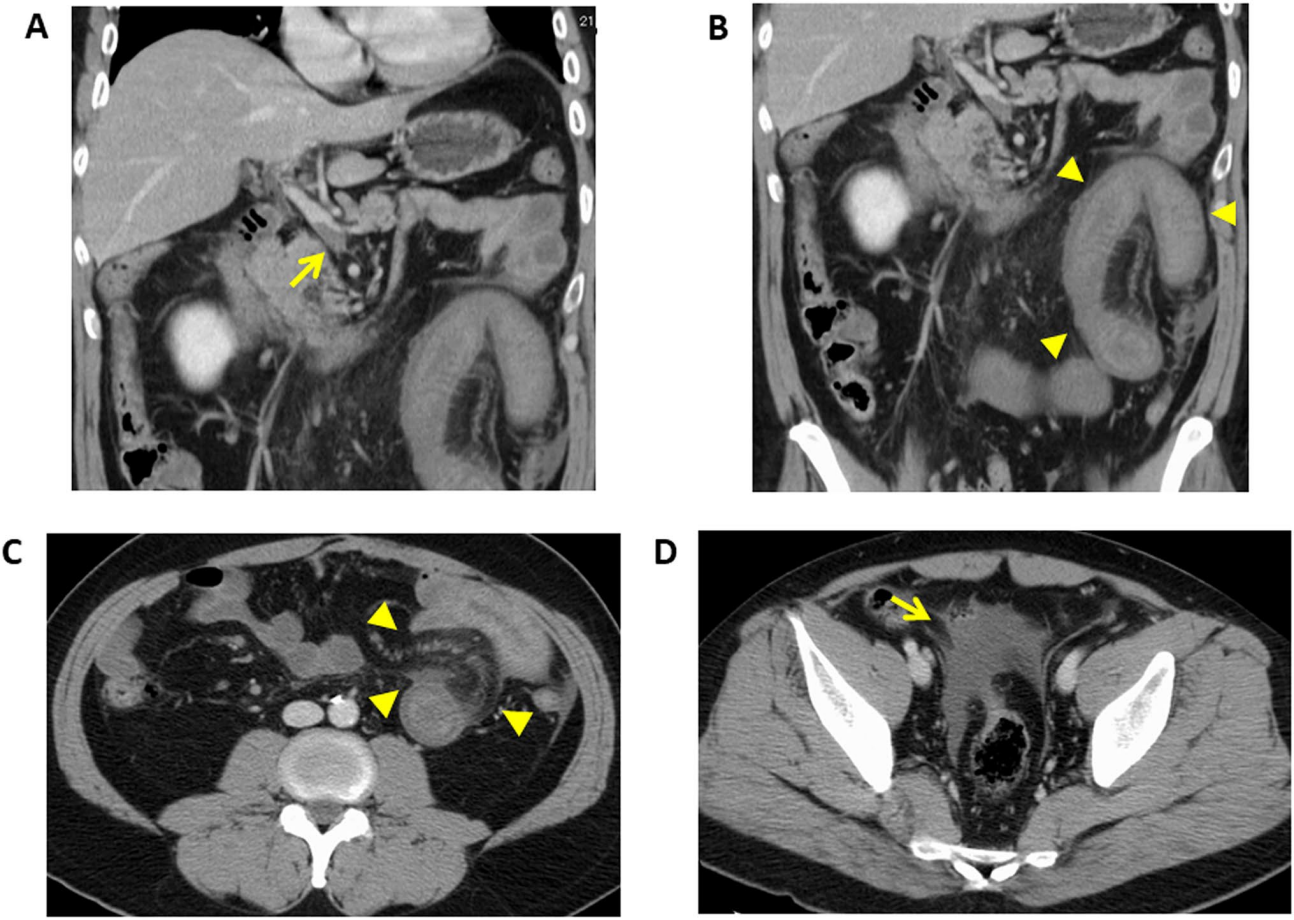
Mesenteric venous thrombosis in a 45-year-old man with a history of splenectomy for idiopathic thrombocytopenic purpura. **A** Coronal contrast-enhanced CT image showing thrombi in the superior mesenteric vein (arrow). **B** The bowel wall is thickened and a target or halo appearance is noted (arrow heads). Bowel resection revealed severe edema and scattered hemorrhages involving all layers. Pathology revealed necrotic changes confined to the mucosa. **C** Contrast-enhanced CT image. Note mesenteric stranding (arrow heads). **D** Contrast-enhanced CT image. The arrow points to ascites

Bowel-wall thickening and hyperenhancement due to hyperemia of the bowel are also seen in venous ischemia [9, 22] (Fig. 8). The presence of venous obstruction can help to differentiate between venous ischemia and reperfusion. Mesenteric edema and fluid may be more frequent in venous than in arterial ischemia (Fig. 8) [5, 9].

### Findings due to bowel obstruction

In patients with bowel obstruction, the venous outflow and then the arterial inflow are obstructed [8]. Consequently, the degree of bowel-wall enhancement varies and depends on the obstruction duration. In the early phase of bowel obstruction, only the venous outflow is compromised and hyperenhancement may be observed. Prolonged enhancement can be seen due to a reduction in arterial perfusion and venous outflow [9, 22]. When the arterial supply is decreased, sub-acute findings are diminished and there may be no mural enhancement (Fig. 4) [5]. Like arterial ischemia, mesenteric stranding is predictive of ischemia in small bowel obstruction (Fig. 4) [31].

## Factors involved in the development of small bowel ischemia (Table 2)

**Table 2:** Clinical features and typical CT findings in patients with small bowel ischemia and in each cause

Factor		Acute mesenteric ischemia			Small bowel obstruction
		Acute mesenteric arterial occlusion	Non-occlusive mesenteric ischemia	Mesenteric venous thrombosis	
Cause		Arterial embolism or thrombosis	Severe vasospasm of the mesenteric artery	Venous thrombosis	Adhesion, hernia, and malignant tumors
Mechanisms in bowel ischemia		Arterial ischemia	Arterial ischemia	Venous ischemia	Increased luminal pressure
Incidence in all emergency room admissions		0.09–0.20%			15%
Incidence in acute mesenteric ischemia		75%	5–15%	5–15%	Not applicable
Presentation		Embolism: acute, thrombosis: sub-acute	Acute or sub-acute	Sub-acute	Acute
Typical CT imaging findings	Bowel	Paper-thin without reperfusion, thickening with reperfusion	Paper-thin without reperfusion, thickening with reperfusion	Thickening	Depends on the duration of obstruction
	Others	Emboli or thrombi in the mesenteric arteries	Narrowing of the diameter of the superior mesenteric artery	Thrombi in the mesenteric vein	Depends on the cause(s)
Treatment		Embolectomy or thrombolysis, surgery for non-viable bowel	Treat cause first, papaverine, resect non-viable bowel	Anticoagulation therapy, resect non-viable bowel	Surgery for strangulated bowel and its cause if needed

### Acute mesenteric ischemia

Acute mesenteric ischemia can be defined as a sudden interruption in the blood supply to a segment of the small intestine. If untreated, it can result in ischemia, cellular damage, intestinal necrosis, and eventual death. It is uncommon in patients reporting abdominal pain and its overall incidence is low; only 0.09–0.20% of all acute admissions to emergency rooms are due to acute mesenteric ischemia. A prompt diagnosis and intervention are essential to reduce its high mortality rate (50–80%) [2]. Mesenteric arterial occlusion, mesenteric venous thrombosis, and non-occlusive mesenteric ischemia are factors involved in the development of acute mesenteric ischemia. Of these, mesenteric arterial occlusion and non-occlusive mesenteric ischemia can induce arterial bowel ischemia while venous ischemia can occur in patients with mesenteric venous thrombosis.

#### Acute mesenteric arterial occlusion

Acute mesenteric arterial occlusion can be due to arterial embolism or thrombosis. Among patients with acute mesenteric ischemia, 50% present with arterial emboli [2]. Emboli arising at the left atrium are associated with cardiac dysrhythmias such as atrial fibrillation, a poor ejection fraction from the left ventricle, or endocarditis affecting the cardiac valves. The emboli typically lodge at sites of normal anatomic narrowing. The superior mesenteric artery (SMA) is particularly vulnerable because of its relatively large diameter and low takeoff angle from the aorta. As most emboli are lodged 3–10 cm distal from the origin of the SMA, the proximal jejunum and colon tend to be spared [32]. More than 20% of SMA emboli are associated with concurrent emboli in another arterial bed in the spleen or kidney [2]. Symptom onset is often acute, severe, and rapidly progressing due to the poor development of collateral vessels. Acute mesenteric arterial thrombosis, seen in approximately 25% of patients with acute mesenteric ischemia, is usually associated with pre-existing chronic atherosclerotic disease leading to stenosis. Thrombi usually arise at the origin of visceral arteries. In the course of years, a plaque in the SMA tends to progress to critical stenosis resulting in collateral beds. Consequently, unlike in patients with acute mesenteric arterial embolism, the presentation can be sub-acute in patients with acute mesenteric arterial thrombosis. As many patients with acute mesenteric arterial thrombosis have a history consistent with chronic mesenteric ischemia including postprandial pain, weight loss, or “food fear”, their history must be examined systematically when acute mesenteric ischemia is suspected [2].

**Fig. 9:**
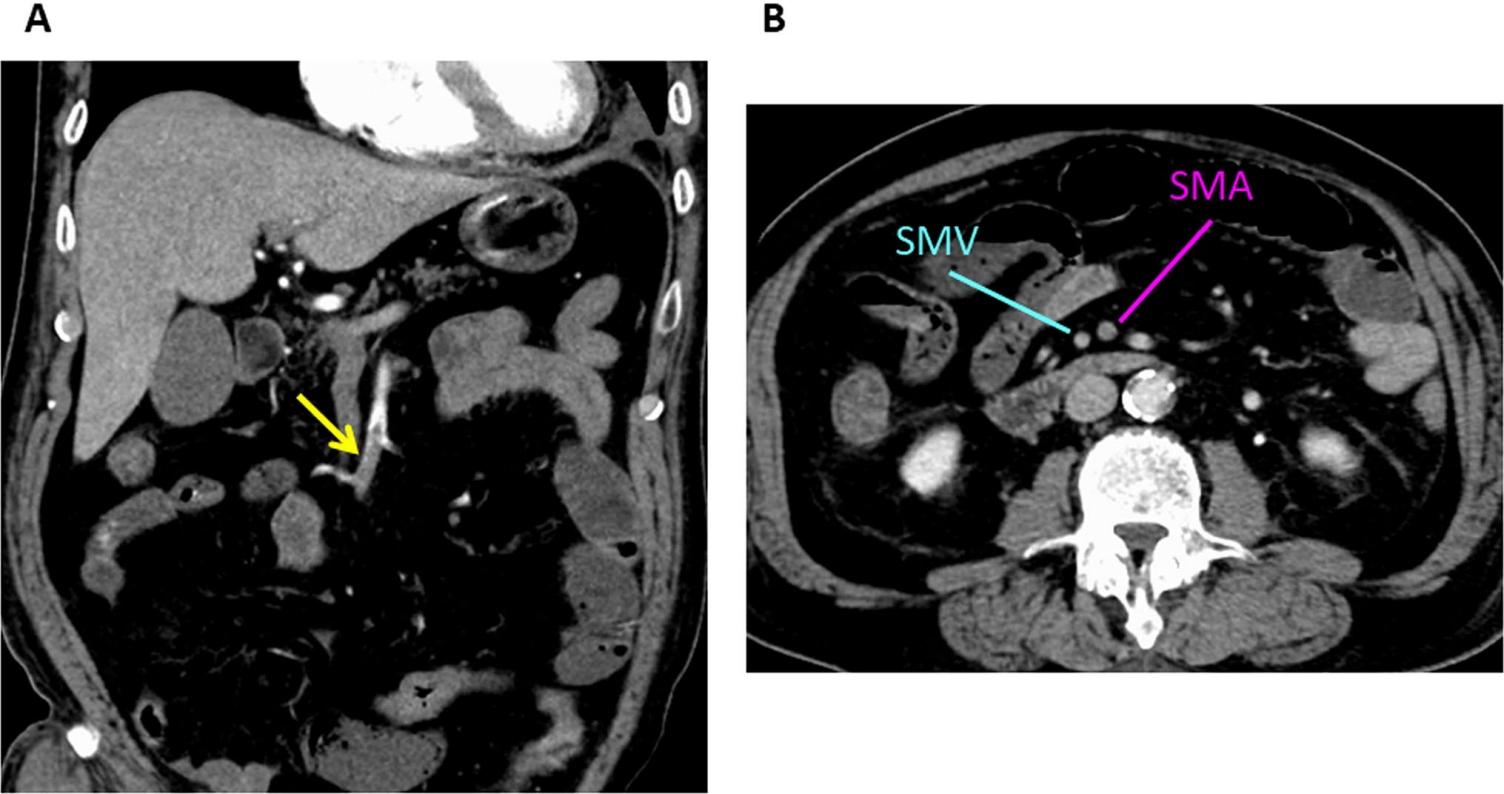
Superior mesenteric artery embolism in a 66-year-old man immediately after surgery for left lung cancer. **A** Coronal contrast-enhanced CT image (arterial phase) showing an occluding embolus (arrow) in the superior mesenteric artery. **B** On the contrast-enhanced CT image (venous phase), the diameter of the superior mesenteric vein (SMV) is smaller than that of the superior mesenteric artery (SMA)

In most instances, emboli or thrombi in the mesenteric arteries are clearly depicted on contrast-enhanced CT images (Figs. 5 and 9). On unenhanced CT images, SMA emboli can be highly attenuated. In many patients with acute superior mesenteric occlusion, the diameter of the superior mesenteric vein (SMV) is smaller than the diameter of the SMA. Hayakawa et al. [33] called this ‘the smaller SMV sign’ and posited that it was attributable to a decrease in the venous return due to blockage of the arterial flow (Fig. 9). Bowel ischemia due to acute mesenteric arterial embolism and thrombosis is elicited by inflow obstruction. Thus, contrast enhancement of the bowel wall is absent or diminished because of the decrease or cessation of the arterial supply. When bowel ischemia progresses to transmural infarction, the bowel wall appears paper-thin. In the case of reperfusion or rich collaterals, the involved bowel segments may become thick and show a halo or target pattern on contrast-enhanced CT images. The clinical significance of reperfusion depends on whether it is effective or ineffective.

The mortality rate is high (60–80%) [34]. Peritonitis secondary to bowel necrosis mandates immediate surgery. The goal of surgical intervention is to re-establish the blood supply to the ischemic bowel, the resection of all non-viable bowel segments, and the preservation of all viable bowel. Endovascular embolectomy can be performed by percutaneous mechanical aspiration or thrombolysis that permits percutaneous transluminal angioplasty. In patients with acute partial or complete occlusion of the SMA, stents may or may not be needed. Any evidence of bowel ischemia or infarction precludes the use of thrombolytic therapy [2].

#### Non-occlusive mesenteric ischemia

Non-occlusive mesenteric ischemia (NOMI), defined as intestinal hypoperfusion in the absence of vascular occlusion, is seen in 5–15% of all patients with acute mesenteric ischemia; its mortality rate is approximately 50%. NOMI is usually seen in patients with debilitating comorbidities such as shock, cardiac disorders, post-operative stress, pancreatitis, burns, dehydration, and hypovolemia [35]. Cardiac surgery, dialysis, and long-term extracorporeal circulation are risk factors for NOMI. The pathophysiological correlates are vasoconstriction and severe vasospasm of the mesenteric arteries that are thought to be attributable to a combination of low cardiac output and vasoconstriction.

A 50% decrease in the blood flow through the SMA triggers autoregulation, and compensatory vasodilation develops in the splanchnic circulation. When hypoperfusion lasts for several hours, it ceases to be effective; the consequence is mesenteric vasoconstriction. However, this situation is reversible if the decline in the flow through the SMA is corrected promptly. When splanchnic vasoconstriction lasts more than 30 min, it is irreversible even after total reestablishment of the arterial blood flow [36]. NOMI patients usually present with sudden-onset abdominal pain, abdominal distension and, in the advanced stage, signs of peritonitis. The prognosis of patients with NOMI is unfavorable because their symptoms are usually masked by sedatives and/or analgesics [37].

Invasive digital subtraction angiography (DSA) is the gold standard for diagnosing NOMI because it demonstrates signs like a blood flow reduction, areas of vessel narrowing and spasm, and consecutive irregularities in the mesenteric vessels [38, 39]. Although angiographic studies of the SMA are essential for the diagnosis of NOMI, they cannot be performed in patients in poor or unstable condition. For the evaluation of NOMI, the diagnostic performance of MDCT angiography is comparable to DSA [40, 41] (Fig. 7). In addition, the diameter of the SMA on CT images was smaller in NOMI patients than in their normal controls [42]. As NOMI is commonly due to splanchnic hypoperfusion and the flow in the SMA is decreased, the return flow in the SMV may be decreased. Nakamura et al. [43] also reported that on CT scans, the diameter of the SMV was smaller in NOMI patients than in the normal controls. This suggests that measuring the diameter of the SMV and the SMA contributes to diagnosing NOMI on CT images. Bowel ischemia in NOMI is the result of inflow obstruction. Thus, as in mesenteric arterial occlusion, the bowel wall in NOMI becomes thinner in the advanced stages with severe ischemia (Fig. 6). After arterial reperfusion, it is thick with a target or halo appearance or shows hyperenhancement on contrast-enhanced CT images. As in patients with mesenteric arterial occlusion, the clinical significance of reperfusion depends on its effectiveness or ineffectiveness. In NOMI, the portion of the involved bowel is usually broad and includes the small intestine and colon; characteristically it is discontinuous and segmental with intervening normal bowel [29].

In the management of NOMI, the underlying precipitating factor(s) are treated when possible to facilitate mesenteric perfusion. Fluid resuscitation, optimization of the cardiac output, and the elimination of vasopressors are important primary measures. Additional treatments may include systemic anticoagulation therapy and direct catheter infusion of vasodilatory and antispasmodic agents; the most commonly administered drug is papaverine hydrochloride [44]. In patients with peritoneal signs, exploratory laparotomy must be performed to resect frankly necrotic bowel portions; however, the condition is often critical and the mortality rate is very high (50–85%) [45]. Damage-control mode, the surgical modality of choice in critically ill patients with acute mesenteric ischemia for physiological and technical reasons, is an important adjunct given the critical condition of these patients [2].

#### Mesenteric venous thrombosis

In 5–15% of patients with acute mesenteric ischemia, mesenteric venous thrombosis is observed. Mesenteric venous thrombosis is secondary to underlying diseases such as portal hypertension, hypercoagulation due to protein C- and protein S-deficiency, polycythemia, a factor V Leiden mutation, right-sided heart failure, abdominal trauma, abdominal infection, acute pancreatitis, malignancies, nephrotic syndrome, and cirrhosis which are contributing factors in 50–75% of all mesenteric venous thrombosis patients [9, 46, 47]. Oral contraceptives, pregnancy, and puerperium are risk factors in young women. Primary mesenteric venous thrombosis without an underlying disease is seen in fewer than 30% of patients [46]. Unlike acute mesenteric arterial occlusion and NOMI, the onset of mesenteric venous thrombosis is characterized by sub-acute abdominal pain that may appear in the course of 2–4 weeks; patients may suffer nausea and vomiting. Even in patients with an extensive clot burden, ischemia develops more gradually than in patients with arterial acute mesenteric arterial occlusion or NOMI and they typically do not suffer infarction unless there is extensive involvement of the upstream peripheral arcade or the vasa recta branches [48].

As thrombi in the mesenteric veins are clearly visualized on contrast-enhanced CT images (Fig. 8), such studies are essential for a diagnosis of acute mesenteric venous embolism. Engorgement of the mesenteric veins due to congestion in the venous outflow is typically seen [9]. Bowel ischemia due to mesenteric venous thrombosis is elicited by outflow obstruction and bowel-wall thickening (Fig. 8), fat stranding in the mesentery and ascites are common findings in patients with mesenteric venous thrombosis (Fig. 8). The bowel typically features a halo or target pattern of enhancement. In patients with severe ischemia, there is no or diminished bowel wall enhancement. It is important to recognize that in mesenteric venous thrombosis, the degree of bowel-wall thickening, mesenteric fat stranding, or ascites does not reflect the severity of ischemic bowel damage because it is due to outflow rather than inflow obstruction [29].

In general, the outcome in patients with mesenteric venous thrombosis, whose mortality rate is approximately 44%, is better than in patients with arterial ischemia such as mesenteric arterial occlusion and NOMI [45]. Non-operative management should be considered in mesenteric venous thrombosis patients without peritonitis. The first-line treatment is anticoagulation therapy. When clinical signs demand surgical intervention, only the obviously necrotic bowel segment(s) should be resected and damage-control measures should be applied. Anticoagulation therapy may improve the clinical picture over the ensuing 24–48 h. The early administration of heparin has been associated with improved survival rates [2, 49].

### Small bowel obstruction

Small bowel obstruction is an important etiology of bowel ischemia. It is due to intrinsic or extrinsic processes that can be benign or malignant. The most common factors are adhesion, hernia, and malignant tumors [50]. Although the overall incidence of bowel obstruction is not known, 15% of all emergency room admissions related to abdominal pain are due to small bowel obstruction [51, 52].

Small bowel obstruction can cause the material inside the bowel to back up into the stomach, resulting in nausea and vomiting of dark green bile (bilious vomiting). The bowel preceding the obstruction becomes large, dilated, and filled with the fluid and air that would otherwise move forward. This elicits bloating (abdominal distention) [53]. In patients with small bowel obstruction, imaging is performed to achieve four goals: (a) determine whether small bowel obstruction is present, (b) locate the obstruction, (c) identify the cause, and (d) look for complications such as strangulation or perforation. The degree of bowel wall enhancement varies and depends on the obstruction duration. Mesenteric stranding is predictive of ischemia in small bowel obstruction (Fig. 4) [31]. Careful evaluation is needed not only to look for complications, but also for implicated factors because some implicated factors (e.g., closed-loop obstruction, incarcerated hernia, and small bowel tumor) are unlikely to be addressed without surgery.

Strangulation is observed in 5–42% of patients with small bowel obstruction [54]; their mortality rate is high (20–37%) [55]. Thus, immediate surgery is recommended in patients with strangulation, bowel ischemia, and/or peritonitis; bowel necrosis contraindicates laparoscopic resection [4, 56, 57]. Patients without strangulation can be managed without surgery. The overall success rate of non-operative management of patients with adhesive small bowel obstruction is 65–83% [58, 59]. However, this type of management is associated with higher recurrence rates and lower disease-free intervals than surgical treatment [60–62]. On the other hand, small bowel obstruction caused by surgically correctable causes (e.g., closed-loop obstruction, volvulus, intussusception, incarcerated hernia, gallstone ileus, foreign body ingestion, and small bowel tumor) is not likely to resolve without surgery. Therefore, surgery is generally offered to these conditions although surgical exploration does not need to be "immediate" as it is for bowel strangulation [63].

## Conclusion

Acute small bowel ischemia has a high mortality rate and requires immediate intervention to avoid unfavorable outcomes. As clinical and laboratory signs of bowel strangulation lack sensitivity for predicting bowel ischemia, CT imaging studies are important for treatment decisions. Small bowel ischemia can be induced by arterial ischemia such as acute mesenteric arterial occlusion and NOMI, venous ischemia including mesenteric venous thrombosis, and increased luminal pressure in the setting of small bowel obstruction. CT findings suggesting bowel ischemia vary depending on the factors eliciting arterial ischemia, venous ischemia, and increased luminal pressure. Reperfusion may alter the findings in patients with arterial bowel ischemia. Therefore, to reach accurate treatment decisions, the correct interpretation of CT images requires an understanding of the factor(s) implicated in the development of small bowel ischemia.

## Data Availability

The data that support the fndings of this study are available on request from the corresponding author, [YN]. The data are not publicly available due to their containing information that could compromise the privacy of research participants.
